# An index system for evaluating the data security in trauma centers: a modified Delphi study in China

**DOI:** 10.1515/jtim-2026-0063

**Published:** 2026-08-26

**Authors:** Feifei Jin, Guozheng Cao, Wei Huang, Tianbing Wang

**Affiliations:** Trauma Medicine Center, Peking University People's Hospital, Beijing, China; Key Laboratory of Trauma treatment and Neural Regeneration, Peking University, Ministry of Education, Beijing, China

**Keywords:** trauma, data security index, Delphi method, analytic hierarchy process, weight analysis

## Abstract

**Background and Objectives:**

In response to the characteristics of frequent data interactions between pre-hospital emergency care and multi-departmental in-hospital systems, along with complex transmission pathways, we established a dynamic assessment methodology covering the entire data lifecycle and analyzed the status of data security risks within China's trauma care system. This study aims to develop a security risk assessment index for trauma data security (TDSI).

**Methods:**

Evaluation indicators were established through qualitative interviews, literature reviews, and Delphi expert consultations. The weight for indicators was determined using the analytic hierarchy process (AHP). National surveillance employing a multi-stage random cluster sampling method was conducted at 80 trauma centers in 31 provincial-level administrative units in China in 2023. The Delphi expert consultation recruited administrators, clinicians, and data specialists from trauma centers across various regions of China. Twenty experts from various regions of China were recruited for the Delphi study.

**Results:**

The response rate for the final survey was 95%. The TDSI system consisted of three dimensions and 36 indicators. The weight was 0.388 for trauma care data management from the clinical department, 0.342 for information security technologies from the IT department, and 0.270 for data security policies from the management department. The overall TDSI across all trauma centers was 60.27, varying from 25.7 to 82.1 across trauma centers. The northern region had the highest TDSI (66.32).

**Conclusions:**

This study developed a TDSI using a modified Delphi method. This index system is a scoring framework that focuses on clinical, management, and IT capabilities that determine the prioritization of data security enhancements for trauma centers.

## Introduction

Trauma is a critical global issue, causing 5.8 million deaths each year and ranking fifth in global causes of death,^[1, 2, 3]^ and more than 60 million people were hospitalized due to severe trauma in China.^[4, 5, 6, 7]^ Despite advancements in trauma emergency technology, mortality and disability rates for severe trauma in China are still significantly higher than in developed countries.^[8, 9, 10, 11, 12]^ To improve the outcomes for trauma patients, trauma centers in China are committed to reducing pre-hospital transport times and in-hospital preparation times for patient reception and utilizing information technology to rapidly transmit patients' medical information.^[13]^

However, because trauma care involves pre-hospital transport and in-hospital emergency treatment, it often requires the cooperation of different departments. Trauma data originate from diverse sources, and the transmission process is complex, posing greater challenges to the security of trauma data.^[14, 15, 16]^

Risk control for trauma data security not only requires IT-based technical safeguards, but also necessitates policy management within hospitals and the standardization of trauma care processes. Long-term and sustained monitoring and governance require action at the global and national levels. Before conducting a nationwide trauma center data security governance, it is necessary to establish a monitoring system and related indicators to assess the security of trauma data and its changes.

Medical data security has become a key focus for the national government. The US Department of Health and Human Services (HHS) released the “Draft Federal Health IT Strategic Plan 2024-2030,” aimed at advancing the development and application of medical information technology and assisting healthcare organizations in implementing effective cybersecurity measures.^[17]^ The European Union has also issued the General Data Protection Regulation (GDPR) and the European Health Data Space legislation.^[18,19]^ The Chinese government has issued the Data Security Law and the Personal Information Protection Law.^[20,21]^ It is necessary to monitor and evaluate the data security associated with trauma to gain insights into the state of data security in trauma centers nationwide, thereby identifying high-risk hospitals and regions. This study aimed to construct a comprehensive index for trauma data security (TDSI) in China.

## Methods

### Developing the preliminary indicator pool

This study uses qualitative interviews, the Delphi method, and an analytic hierarchy process (AHP) to build a comprehensive index for trauma data security. Qualitative interviews can provide in-depth insights into suggestions for different roles. The Delphi method is a structured process and a systematic, effective, reliable, and comprehensive technique for collecting and distilling knowledge from a group of experts using a series of anonymous questionnaires interspersed with controlled feedback.^[22, 23, 24, 25]^

**Figure 1:**
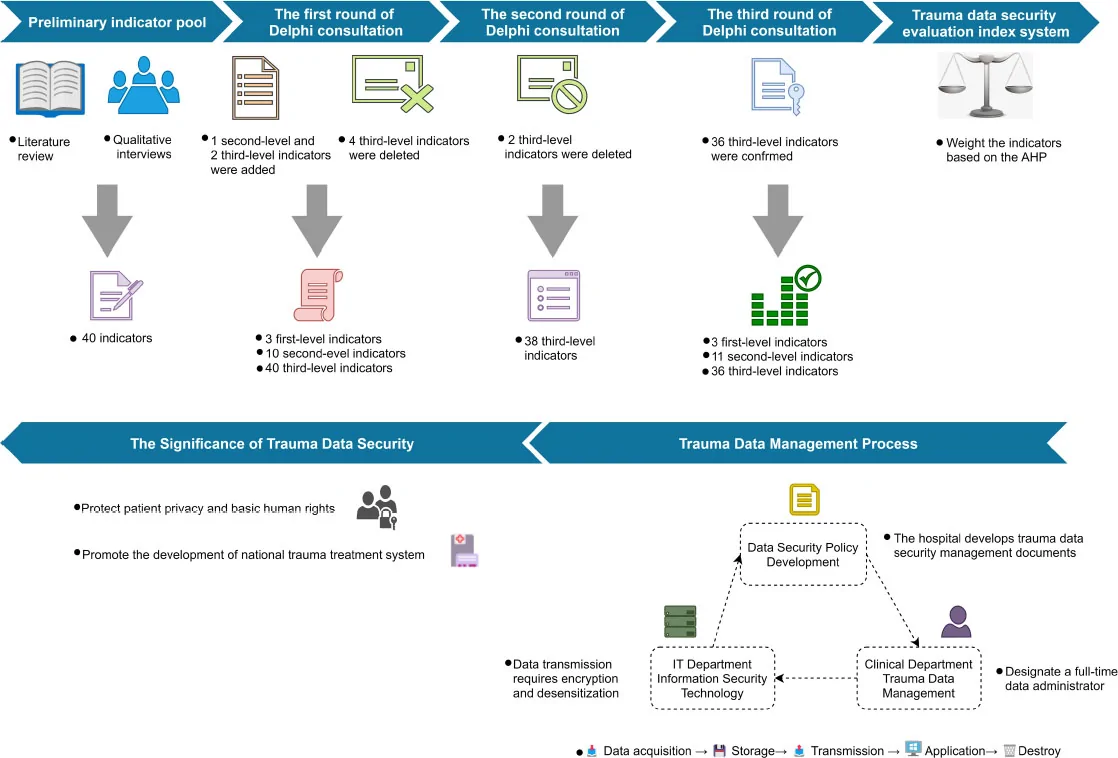
Study overview. AHP: analytic hierarchy process.

The AHP method was used to perform weight calculations and assignments for each indicator.^[26, 27, 28]^ The procedure used in this study is illustrated in Figure 1.

### Defining dimensions for TDSI

First, according to the medical data security regulations in various regions of China as well as those obtained from the literature review, we concentrate on data management processes, information technology, and data security policies. Secondary and tertiary indicators are determined for each dimension. Then, face-to-face, semi-structured qualitative interviews were conducted to gather diverse perspectives on dimensions and indicators for trauma data security, and the relevant findings were published.^[29]^ Finally, we defined three dimensions for TDSI and established an indicator database.

### Index system construction

The Delphi method involves gathering expert opinions using a structured process.^[30, 31, 32]^ The steps include organizing the events into a rating table, creating and distributing a Delphi questionnaire, collecting and analyzing expert feedback, and iteratively refining the feedback until a consensus was reached.^[33,34]^ First, we identified the indicators for discussion through a systematic review of the relevant literature. We then conducted three rounds of Delphi consultations on the selected indicators to achieve expert consensus. Beginning with the second round, we provided experts with both a questionnaire and a summary of the results of the previous round, allowing them to reconsider and revise their judgments to determine the final set of indicators. Figure 1 illustrates this process.

#### A Expert selection

The experts were selected based on criteria such as having at least an intermediate professional title, extensive experience in assessing medical data security, and familiarity with hospital trauma care processes. Experts from medical institutions and universities were also included.

#### B Consultation

The consultation questionnaire describes the research background, objectives, significance, and scoring criteria. The authority of their opinions was measured using the authoritative coefficient (Cr), which is the average of the judgment basis (Ca) and familiarity with the indicators (Cs). A Cr value of 0.7 or higher was deemed reliable. Familiarity (Cs) was self-assessed and categorized into five levels with corresponding weights. The judgment basis (Ca) was evaluated across four dimensions: practical experience, theoretical analysis, reference to research, and intuitive perception. Each score was further classified into degrees based on assigned scores. The experts also provided demographic and professional information and suggested modifications or additional indicators.

#### C Data summary

Expert opinions were collected during two rounds of the Delphi. The arithmetic mean (M) represents the concentration of opinions, and the response rate indicates expert engagement. The coordination coefficient (W) measures consensus among experts, with higher W values implying better coordination. The formula for W is *W* = 1 (the sum of the standard deviations/overall variation), where the overall variation is derived from the sum of squared differences between each expert's rating and the mean divided by (*n* − 1).

### Using AHP to assign weights

The AHP method determined the weights for TDSI.^[35, 36, 37]^ Trauma data security evaluation, three primary dimensions, and the corresponding secondary indicators. We applied a 1–9 scale of 1–9 to compare the relative importance of indicators at the same level and developed judgment matrices accordingly. The weight coefficients were then derived by calculating the eigenvectors of these matrices. The weight analysis used a 9-point scale, as detailed in Supplementary Table S1. Fifteen experts scored three levels of the indicators, and the scores were averaged to derive the mean values. This approach established the weights of the indicators within each level of the judgment matrix. The overall weight of the index system is calculated by multiplying the weights of the indicators at each level. The calculation process:

#### A Building a hierarchical structure

The target and indicator layers are defined by an indicator layer, which potentially has multiple sublayers.

#### B Creating a judgment matrix

A judgment matrix was formed using the target and indicator layers. The resulting matrix is as follows:


$$U=\left[\begin{array}{cccc} a_{11} & a_{12} & \ldots & a_{1n}\\ a_{21} & a_{22} & \ldots & a_{2n}\\ \vdots & \vdots & \ddots & \vdots \\ a_{n1} & a_{n2} & \ldots & a_{nn} \end{array}\right]$$

#### C Calculating weights using the judgment matrix

The weights were calculated using the geometric mean method. Initially, a new column vector was formed by multiplying the rows. Each element of this vector was then raised to the power of “n” based on the order. Finally, the vector is normalized column-wise to yield a weighted vector, as shown in the following formula:


$$\omega_{i}=\frac{{\left(\prod_{j=1}^{n}a_{ij}\right)}^{\frac{1}{n}}}{\sum_{k=1}^{n}{\left(\prod_{j=1}^{n}a_{kj}\right)}^{\frac{1}{n}}},(i=1,2,\ldots ,n)$$

#### D Consistency assessment

Given the subjective nature of hierarchical analysis, consistency must be assessed. A random consistency ratio (CR) below 0.1 was considered acceptable. If the CR exceeds this threshold, adjustments are necessary to improve consistency. The formula used were:


$$CR=\frac{CI}{RI}$$

$$CI=\frac{\lambda_{\max}-n}{n-1}$$

$$\lambda_{\max}=\frac{1}{n}\sum_{i=1}^{n}\frac{(A\omega {)}_{i}}{\omega_{i}}$$

where *CI* is the consistency index, *RI* is the random index (Supplementary Table S2), and *A* represents the judgment matrix value.

#### E Calculating the final index weights

The final weight of each indicator system was determined by multiplying the weights of the indicators at each level.

### National surveillance

We conducted a cross-sectional survey in the Chinese mainland. The Chinese mainland comprises six geographical regions (north, northeast, east, central and south, southwest, and northwest) encompassing 31 provincial-level administrative units (the Hong Kong Special Administrative Region, Macao Special Administrative Region, and Taiwan Province were excluded). These factors vary in economic development, population size, and geographical features. Medical service quality also differs among provincial, city, and county trauma centers. A multi-stage random cluster sampling method was adopted to select one provincial, one municipal, and one county-level trauma center from each province for better representativeness. Eighty hospitals completed the survey, covering 31 provincial-level administrative units and six geographical regions of China. There were 25 provincial-, 28 municipal-, and 27 county-level trauma centers. Participating trauma centers were included based on representativeness and feasibility. This study was designed to achieve coverage across different geographical regions and institutional tiers of trauma centers in the Chinese mainland. Centers were eligible if they met the established criteria for trauma center designation and if they agreed to participate in the survey. All eligible centers that were successfully contacted and provided consent were included. No additional eligible centers were excluded during data collection. A questionnaire was developed based on TDSI, and survey teams were formed to conduct investigations at each trauma center. The members of the survey teams underwent unified training to standardize the survey process and evaluation criteria. After the survey teams were assessed, each indicator in the questionnaire was scored individually. Each indicator was evaluated using a dichotomous scale (1 = criterion met; 0 = criterion not met), based on a standardized on-site assessment and documentation review. The total TDSI score was calculated as the weighted sum of all indicators, with weights determined by the analytic hierarchy process (AHP).

### Patient and public involvement

No patient was involved in this study.

## Results

### TDSI dimensions and indicators

TDSI should be an objective and sensitive monitoring tool capable of comprehensively reflecting safety issues associated with trauma data. A review of 23 papers helped identify the dimensions of trauma data security and formulate Level 2 and Level 3 indicators. Qualitative interviews were conducted with 27 participants from 12 provincial-level administrative units (average age: 44.70 ± 8.56 years, average work experience: 18.22 ± 9.5 years). Through a literature review and qualitative interviews, TDSI included three dimensions: data security policies from the management department, trauma care data management from the clinical department, and information security technologies from the IT department; 40 indicators were selected (Supplementary Table S3).

### Screening of indicators

Nineteen experts participated in the Delphi study (average age: 43.53 ± 8.64 years, average professional experience: 16.90 ± 8.27 years, all with intermediate or higher professional titles). Table 1 presents the details of the experts. In the first round, 20 experts were consulted with 19 valid responses (95% response rate). In the second round, the response rate was 100%.

The opinions of experts were highly concentrated. The expert authority coefficient was 0.85, and the coordination coefficient improved from 0.159 to 0.332, with a *P*-value of < 0.05, indicating satisfactory expert agreement (Table 2). According to the principle of indicator screening, after two rounds of the Delphi method, the TDSI included three dimensions and 36 indicators.

**Table 1:** Basic information of Delphi consultation experts for trauma data security risk assessment

Information	Number	Percentage (%)
Age (years)		
< 40	10	52.63
40–49	3	15.79
≥50	6	31.58
Years of professional experience		
< 10	3	15.79
10–19	7	34.84
≥20	9	47.37
Professional title		
Senior	6	31.58
Associate Senior	5	26.32
Intermediate	8	42.11
Education		
Doctoral degree	12	63.16
Master’s degree	5	26.32
Bachelor’s degree	2	10.53
Research field		
Trauma medicine	10	52.63
Information science	6	31.58
Data regulations	3	15.79

**Table 2:** Delphi-related indicators for TDSI

Round	Judgment basis of the indicators (Ca)	Familiarity with the indicators (Cs)	Authoritative Coefficient (Cr)	Positivity coefficient	W
1	0.86	0.78	0.82	95%	0.159
2	0.86	0.89	0.88	100%	0.332

### Index weighting

Fourteen judgment matrices were created. According to the AHP results, the weights for the three main dimensions were as follows: trauma care data management in the clinical department (0.388), information security technologies in the IT department (0.342), and data security policies in the management department (0.270). Within the management department's data security policies, organizational structure (0.548) and management planning (0.452) had the highest weights. For trauma care data management from the clinical department, data transmission (0.302), data application (0.239), data storage (0.173), data capture (0.151), and data destruction (0.134) were ranked. For IT information security, partnership management (0.288), technology security (0.271), environmental security (0.224), and monitoring and auditing (0.216) were weighted. All CR values were below 0.1, indicating high consistency. Table 3 presents the weights at each index level.

**Table 3:** Indices and weights for trauma data security

Dimensions	Sub-dimensions	Indicators	Weight
Data security policies from management department	Management planning Organizational structure	The administrative department of the hospital has formulated a trauma data security management file The Manager in charge of trauma data security is the Director of the Trauma Center; Have a complete trauma data security management team	12.2 5.8 9
Trauma care data management from clinical department	Data capture	Medical staff should not privately record patients' information using mobile phones or paper during the trauma care process; The trauma center has full-time data administrators;	1.2 1
		The collection environment for trauma data meets privacy requirements;	0.8
		Training on data capture security was conducted for medical staff at the trauma center;	1.4
		Medical staff have mastered the classification of security levels for trauma data;	1.4
	Data storage	Medical staff are not allowed to privately store trauma data on personal computers, mobile phones, or other devices;	0.7
		Medical staff are not allowed to privately take un-desensitized trauma data out of the trauma center;	0.7
		All storage medium for trauma data have clear physical identifications;	0.8
		Training on data storage security was conducted for medical staff at the trauma center;	0.8
		The database access in the trauma center has different permission settings;	1.9
		The database content of the trauma center has backups	1.8
	Data transmission	The transmission of trauma data between hospitals will involve signing data confidentiality agreements;	2.3
		The transmission of trauma data is handled by dedicated personnel;	2.2
		Training on data transmission security was conducted for medical staff at the trauma center;	1.4
		Medical staff are not allowed to transmit patients' information through phone calls or text messages;	2.1
		The trauma data need to be encrypted and desensitized before transmission;	2.7
		The remote operating system of the trauma care system has data encryption technology	1.1
	Data application	The use of trauma data is subject to graded access permissions;	2.3
		Training on data application security was conducted for medical staff at the trauma center;	2.9
		The publication of papers and other achievements based on trauma data requires approval from the hospital;	1.7
		Trauma data possess high data quality and can be utilized in clinical research	2.5
	Data destruction	After temporarily using a non-medical record system to document patient data, medical staff will promptly destroy the records;	3.4
		Training on data a destruction security was conducted for medical staff at the trauma center	1.8
Information security technologies from the IT department	Information system partnership management	The information system provider for the trauma center possesses Class III information security protection certification;	5.1
		The hospital and the information system vendors have signed data confidentiality agreements	4.8
	Information system monitoring and auditing	The information system possesses data security monitoring and early warning functions;	3.7
		The information system provider conducts regular security audits;	3.7
	Information system environment security	The operation room of the information system is equipped with monitoring facilities;	3.8
		The operation room of the information system has facilities for preventing natural disasters	3.8
	Information system technology security	The information system is capable of automatically identifying sensitive data;	1.9
		The information system is equipped with security technologies such as anti-hacking measures, firewalls, and antivirus protection;	1.9
		The information system possesses data backup and data recovery technologies;	2.2
		The information system has a complete and tamper-proof operation log	3.2

### National surveillance results of TDSI

**Figure 2:**
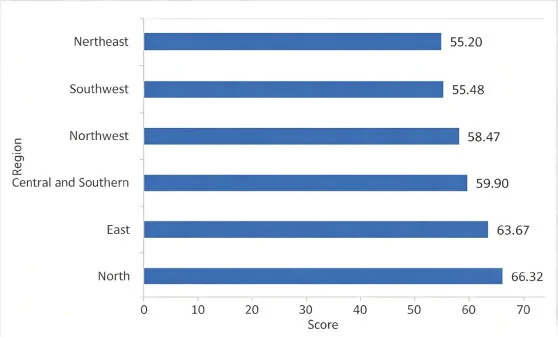
The TDSI for the six geographical regions.

The TDSI average score in China was 60.27 and a provincial trauma center scored 82.1 (highest), while a county-level center scored 25.7 (lowest). The average score for provincial trauma centers was 77.05, for municipal trauma centers was 55.93, and for county-level trauma centers was 48.97. The average scores for the six geographical regions are shown in Figure 2. The northern region had the highest TDSI (66.32) and the northeastern region had the lowest TDSI (55.20).

**Figure 3:**
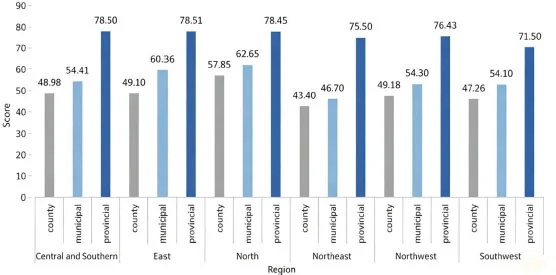
The TDSI for the trauma centers.

Figure 3 shows the average TDSI results for trauma centers at different levels across the six regions. The ranking for TDSI in different dimensions is shown in Supplementary Figure S1-S3, respectively.

### Challenges and solutions for trauma data security

The dynamic nature of trauma data (which often involves rapid collection, transmission, and analysis) requires real-time security measures that do not hinder operational efficiency. Ensuring the security of data capture environments, real-time data transmission, and immediate accessibility without compromising confidentiality and integrity are complex tasks. Supplementary Table S4 outlines the challenges faced by each TDSI indicator in real-time applications, as well as recommended solutions to address these challenges. Our secure TDSI for trauma data systems addresses the challenges of flexible management plans, secure data-handling protocols, and advanced monitoring tools. The solutions include end-to-end encryption, role-based access controls, comprehensive training, and real-time incident response teams. This approach ensures robust protection and reliability throughout the lifecycle of the trauma data.

## Discussion

This study established a TDSI for trauma data security and conducted a nationwide multicenter cross-sectional survey incorporating trauma centers of various levels from 80 hospitals, covering 31 provincial-level administrative units across China. Two rounds of the modified Delphi and AHP methods were used to determine the index and weights of each indicator, and the TDSI system was finally established with the derived weights of the 36 indicators.

### Differences in TDSI across geographical regions and tiered trauma centers

Across the six geographical regions, the northern region had the highest average TDSI score (66.32), whereas the northeastern region had the lowest (55.20). The overall ranking of the six geographical regions was as follows: northern > eastern > central-southern > northwestern > southwestern > northeastern. The relatively high scores observed in the northern and eastern regions may have been associated with structural and institutional factors. The National Trauma Center is located in Beijing within the northern geographical region, and trauma centers in this region maintain close collaboration in data system development and governance practices. During the establishment of trauma databases, institutions in these geographical regions may have aligned more closely with national technical standards and regulatory requirements. In addition, more direct oversight and technical guidance from the National Trauma Center may have contributed to greater standardization of database construction, management procedures, and data supervision practices. These factors may partly explain the differences observed across geographical regions.

From the perspective of institutional tiers, provincial trauma centers achieved the highest TDSI score (82.1), whereas county-level centers achieved the lowest average score (25.7). Across regions, a consistent pattern was observed: provincial > municipal > county-level trauma centers. This pattern may be related to structural differences. First, provincial trauma centers are typically located in economically developed cities with stronger resource allocation capacity. They often operate on a larger scale with more advanced information systems, greater investments in data governance, and more specialized personnel. Second, higher patient volumes and sustained data management experience may also contribute to more standardized operational practices. Finally, provincial trauma centers usually serve as leading data centers within each province and maintain closer collaboration with the National Trauma Center, which may further enhance standardization and oversight. These factors may explain the observed differences between institutional tiers.

### Practical implementation of TDSI

In practical applications, hospitals can use TDSI as a structured reference to examine existing data management practices. By reviewing individual indicators, trauma centers can identify areas where policies remain incomplete, technical safeguards require strengthening, or clinical data procedures require further standardization. In this context, the index supports systematic self-assessment, rather than functioning as a numerical score. Periodic reassessment may also provide insights into whether identified deficiencies have been addressed effectively over time.

At the national surveillance level, aggregated TDSI results can inform regional evaluation and oversight. Comparing institutions of similar levels may reveal shared ideas of weakness, including gaps in encryption practices or inconsistencies in governance procedures. Such findings may assist supervisory authorities in prioritizing areas for improvement. Incorporation of TDSI into existing quality assurance or accreditation frameworks may further enhance its practical relevance and contribute to sustained improvements in trauma data security.

### Interpretation of the trauma data security

The proposed index incorporates multiple dimensions and indicators based on three core concepts: the trauma care data management from clinical department, information security technologies from the IT department and data security policies from management department.^[38, 39, 40]^ The hospital data security policies from management department acts as a crucial support mechanism. The administrative department of the hospital has formulated a trauma data security management file have the greatest impact followed by have a complete trauma data security management team and the manager in charge of trauma data security is the director of the trauma center, highlighting the system's importance and forming the basis for future data security efforts.^[41,42]^

Information security technologies in the IT department include partnership management, monitoring, auditing, environmental security, and technology security, with the importance of supplier qualifications and confidentiality agreements being particularly notable. Therefore, the supplier management requires careful attention.^[43]^

Trauma care data management from the clinical department. In the processes of data collection, transmission, storage, application, and destruction, the behavioral norms of medical staff carry significant weight, which is an important issue identified in this study. During trauma treatment, when patients require urgent medical attention and are transferred between different hospital wards and clinical departments, doctors often resort to mobile phones or paper to record and transmit patient information. Although this practice prioritizes patient rescue, it also poses potential risks to data security. Medical personnel in trauma centers must be trained to fully understand data security and adhere to standardized data management principles during trauma treatment. This may be a common but overlooked phenomenon in hospitals in China and many other countries as more data experts focus on data security technologies related to IT departments.^[44,45]^

### Comparison with relevant studies

Artificial intelligence (AI) techniques are becoming increasingly popular for the development of security data risk assessment tools. For instance, one study used AI and ICT, such as the Advanced Human-Robot Collaboration Model, for safety robot-based risk management in modern workplaces.^[46]^ The advancement of big data and AI offers significant potential for the flexible and efficient development of trauma data security risk assessment tools.

The creation of robust security guidelines requires a thorough approach that integrates various techniques. The literature underscores the use of decision-making frameworks such as fuzzy AHP and the Technique for Order Preference by Similarity to Ideal Solution (AHP-TOPSIS) to evaluate the impact of malware analysis methods on the security of web applications.^[47]^ This process involves critical steps such as detailed manual analysis of deceptive malware, acquiring potential malware datasets, testing them in virtual environments, and creating signatures for malicious programs. Sensitivity analysis plays a crucial role in validating research findings and assessing how changes in factors affect the outcomes to ensure robustness and reliability.

Moreover, it is important to incorporate sustainability characteristics, such as optimizing web-based resources, reducing energy consumption, and ensuring durability, along with traditional security attributes such as confidentiality, integrity, and availability when developing security guidelines.^[48]^ A hybrid fuzzy rule-based multicriteria framework is recommended for assessing sustainable security, considering factors such as sustainability, web-based resource optimization, and portability. Multi-criteria decision-making techniques have also been highlighted to effectively manage conflicting criteria and integrate expert opinions.

Furthermore, effective security guidelines should leverage durability as a security factor, address design coding insecurities, incorporate behavioral aspects into marketing strategies, engage in iterative postemployment security maintenance, and underscore the importance of software security in marketing.^[49]^ To manage uncertainty and the ambiguity inherent in human judgment, fuzzy AHP—which combines fuzzy set theory and AHP—is recommended. Trustworthiness, dependability, and human trust have been identified as key attributes of durable security.^[50, 51, 52]^ By integrating these diverse methods and perspectives, comprehensive and resilient security guidelines can be developed by integrating these diverse methods and perspectives.

In constructing a national trauma data security system for sentinel hospitals, advanced techniques such as fuzzy AHP and fuzzy AHP-TOPSIS, although sophisticated, are unsuitable because of the complexities of gathering trauma data. Instead, the classical AHP method is ideal. Trauma data involve multiple steps and potential vulnerabilities, requiring a structured approach to manage the components effectively. The classical AHP method offers simplicity, transparency, and consistency in decision-making, and is essential for achieving uniform security standards across hospitals. It is easier to implement and adapt, and requires less specialized knowledge than advanced techniques, ensuring smoother adoption by healthcare professionals. Additionally, AHP excels at setting priorities among critical factors. Thus, the structured and straightforward nature of classical AHP makes it suitable for building an effective security assessment system for national trauma data.

### Future work

The TDSI is a pivotal tool for enhancing the security of trauma information systems. We aimed to conduct extensive field testing of TDSI in various trauma centers across China to validate its applicability and effectiveness in diverse settings.^[53, 54, 55]^ We intend to establish a collaborative network among trauma centers to share best practices, and jointly address common security challenges.

### Digital health and trauma data governance

As digital technologies continue to expand in healthcare, trauma data are increasingly generated and managed within interconnected systems. AI tools are now used in clinical settings for functions such as diagnostic support, treatment planning, patient monitoring, and trauma care. Telemedicine-based trauma networks further enable real-time consultation and cross-institutional data exchange, which changes how clinical information is produced and shared.^[56]^

These developments have introduced several practical challenges. The use of multiple information platforms, increased data exchange between institutions, and algorithm-assisted decision making may complicate data management and oversight. In this context, clearer governance arrangements and regulatory guidance are needed to ensure that AI and health information technologies are implemented responsibly.^[57]^ The application of TDSI within defined institutional frameworks may help support data security and ethical standards in trauma care systems.

### Strengths and limitations

This study established a novel dynamic assessment framework (TDSI) for trauma data security that addresses multidepartmental data flow complexities through lifecycle coverage. Methodological rigor was ensured *via* modified Delphi-AHP procedures and national surveillance across 80 trauma centers, revealing actionable regional disparities. The survey covers 31 provincial-level administrative units across the Chinese mainland, representing a broad range of geographical areas and levels of socioeconomic development. The Hong Kong Special Administrative Region, Macao Special Administrative Region, and Taiwan Province were not included in this study because their administrative, legal, and healthcare systems differ from those of the Chinese mainland. Although these three regions were excluded, the provinces operate under a common regulatory and healthcare governance framework. Therefore, these findings are considered representative within the context of the Chinese mainland. Three-dimensional weighting provides prioritized enhancement pathways. However, this study had several limitations. Expert representation lacked urban-rural stratification despite nationwide recruitment. Empirical validation through a large-scale implementation is required to verify TDSI reliability of the TDSI. Standardized assessment tools derived from index needs development and direct patient perspectives on data security were not captured.

The TDSI for China is a comprehensive evaluation system designed to enhance data security in sentinel surveillance hospitals. This system comprised 36 indicators across three dimensions. This index offers a structured framework for evaluating and improving the security of trauma information systems, ensuring comprehensive protection at all stages of data management. Additionally, this study outlines strategies for future use of the index by summarizing potential challenges and proposing solutions for its practical application.

## Supplementary information

Supplementary materials are only available at the official site of the journal (www.intern-med.com).

## Supplementary Material

Supplementary Material Details
