# Supplementary material for: An index system for evaluating the data security in trauma centers: a modified Delphi study in China
Source: J Transl Int Med. 2026 Aug 26;14(4):599–609. doi: 10.1515/jtim-2026-0063 (PMC13509150; doi:10.1515/jtim-2026-0063)
Supplement: Supplementary file 1 [file jtim-2026-0063_sm.pdf]

Supplementary materials

An index system for evaluating the data security in trauma centers: a modified Delphi study in China

Feifei Jin[1,2], Guozheng Cao[1,2], Wei Huang[1,2], Tianbing Wang[1,2]

[1]Trauma Medicine Center, Peking University People's Hospital, Beijing 100033, China

[2]Key Laboratory of Trauma treatment and Neural Regeneration, Peking University, Ministry of Education, Beijing 100871, China

***Address correspondence to:** Tianbing Wang, Trauma Medicine Center, Peking University People's Hospital, No. 11, Xizhimen South Street, Xicheng District, Beijing 100033, China. E-mail: wangtianbing@pkuph.edu.cn; Wei Huang, Trauma Medicine Center, Peking University People's Hospital, No. 11, Xizhimen South Street, Xicheng District, Beijing 100033, China. E-mail: hwei1104@163.com

Supplementary Table S1: Nin-point intensity of the importance scale of the goal tree

**Degree of importance**	**Definition**	**Explanation**
1	Equal importance	Two elements contribute the same to the goal
3	Weak importance	One is slightly more important than the other based on experience and judgment
5	Moderate importance	One is more important than the other based on experience and judgment

7	Strong importance	One is strongly more important than the other, and its dominance is demonstrated in practice
9	Absolute importance	One is absolutely more important than the other
(2,4,6,8)	Intermediate values	Adopted as an option for compromising

Supplementary Table S2: Random index (RI) values

Matrix factor	RI values
3	0.5149
4	0.8931
5	1.1185
6	1.2494
7	1.3450
8	1.4200
9	1.4616

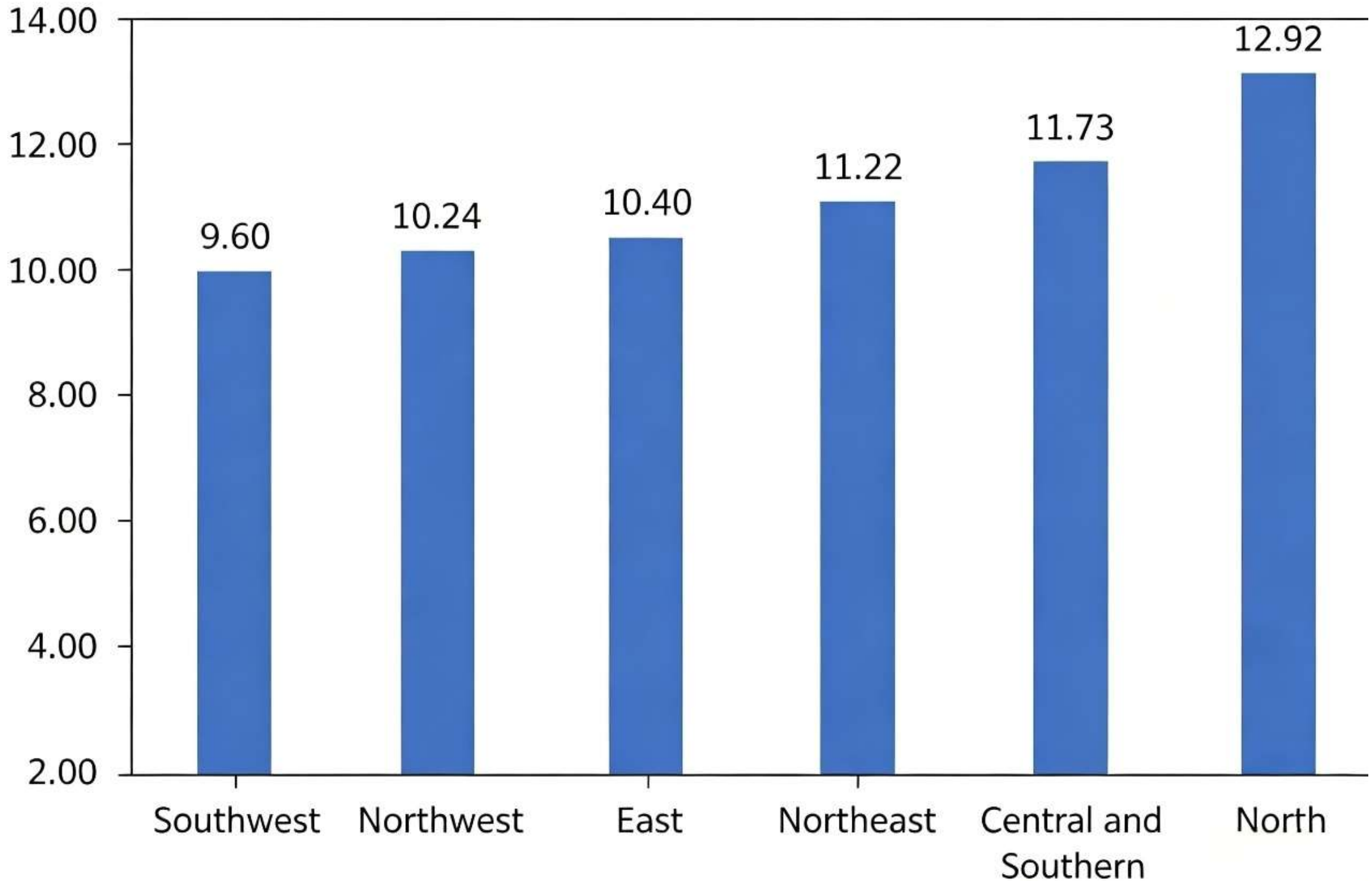

Supplementary Figure S1: The data security policies from management department score for the six geographical regions.

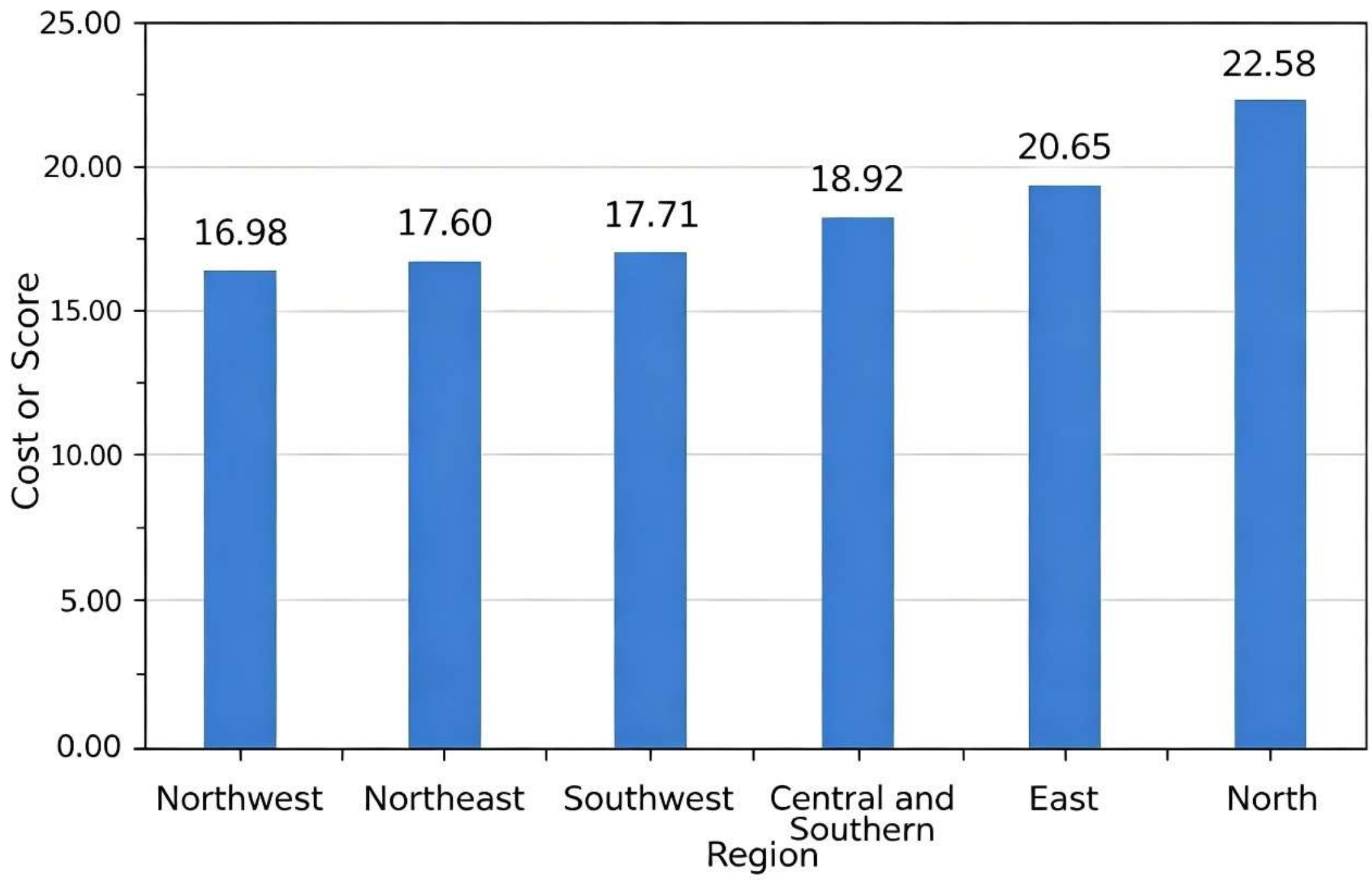

Supplementary Figure S2: The trauma care data management from clinical department score for the six geographical regions.

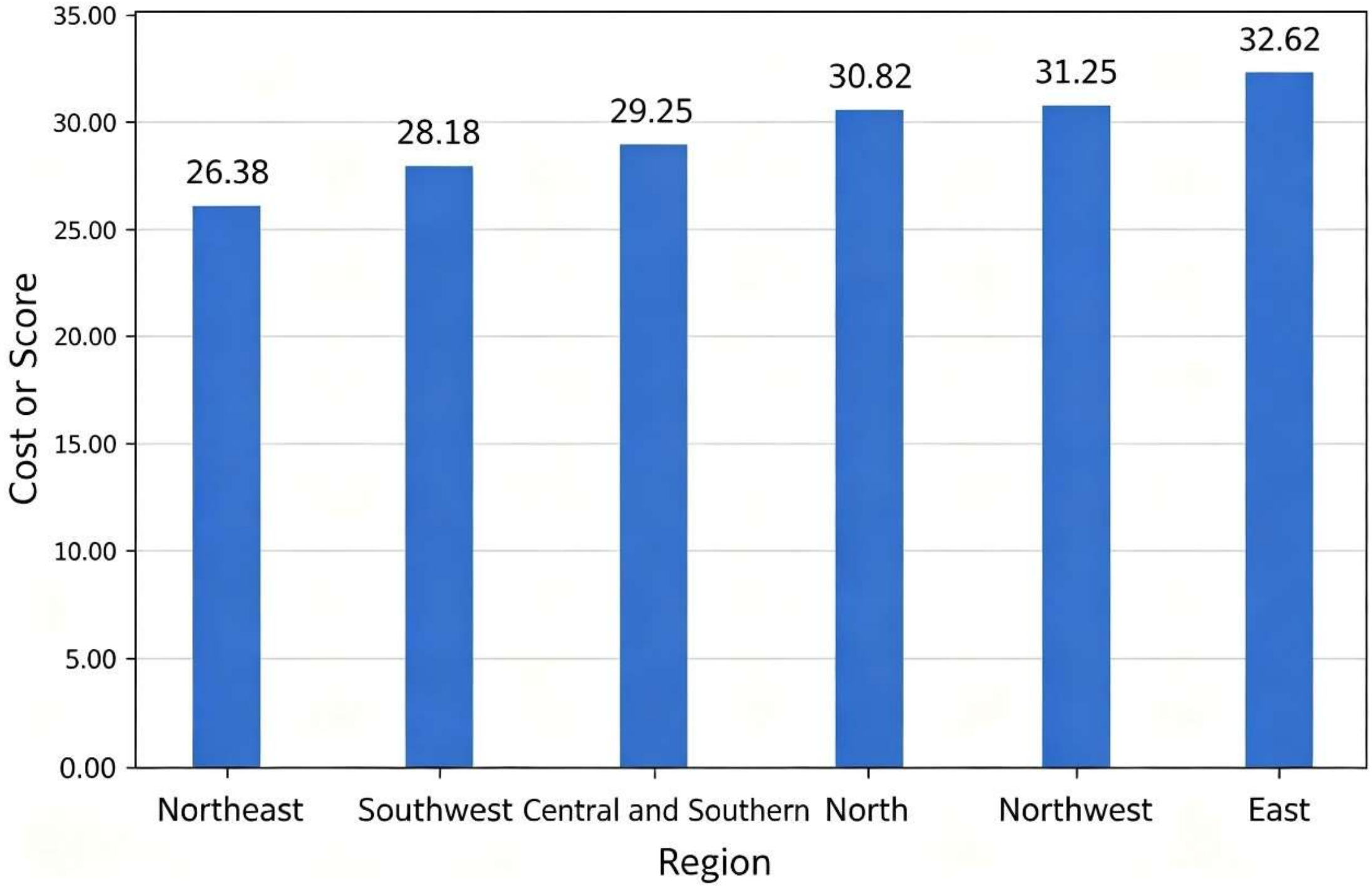

Supplementary Figure S3: The information security technologies from the IT department score for the six geographical regions.

Supplementary Table S3: Preliminary construction of the three-level indicator system for TDSI

Dimension	Sub-dimension	Indicators
1. Data security policies from management department	1.1 Management planning	1.1.1 Hospital policies
	1.2 Organizational structure	1.2.1 Leadership responsibility
		1.2.2 Organizational system
2. Trauma care data management from clinical department	2.1 Data capture	2.1.1 Operational safety of medical personnel
		2.1.2 Dedicated responsibility system
		2.1.3 Data capture environment security
		2.1.4 Data capture security training
		2.1.5 Data capture security knowledge
		2.1.6 Specialized operation system
	2.2 Data storage	2.2.1 Data storage mode

		2.2.2 Data storage sites
		2.2.3 Mobile media identification
		2.2.4 Data storage security training
		2.2.5 Access methods and authorization management
		2.2.6 Database backup
		2.2.7 Management system for USB drives, hard drives, or other storage media
	2.3 Data transmission	2.3.1 Data transmission system
		2.3.2 Dedicated responsibility system
		2.3.3 Data transmission security training
		2.3.4 Data transmission security operations
		2.3.5 Encrypted transmission of business systems
		2.3.6 Remote O&M channel security

		2.3.7 Safety awareness among medical staff
	2.4 Data application	2.4.1 Data access hierarchy
		2.4.2 Data application security training
		2.4.3 Approval of data usage results
		2.4.4 Availability/accessibility
		2.4.5 Institutional documents
	2.5 Data destruction	2.5.1 Operational safety of medical personnel
		2.5.2 Data destruction security training
3. Information security technologies from the IT department	3.1 Information system monitoring and auditing	3.1.1 Monitoring and warning mechanism
		3.1.2 System security audits
	3.2 Information system environment security	3.3.1 Monitoring facilities
		3.3.2 Safety protection facilities

		3.2.3 Convenient transportation
		3.2.4 Natural environment security
	3.3 Information system technology security	3.3.1 Automatic recognition and processing of sensitive data
		3.3.2 Data security protection technology
		3.3.3 System backup and recovery

Supplementary Table S4: Challenges and solutions in real-time applications for trauma data security TDSI

**Level 1 indicators**	**Level 2 indicators**	**Level 3 indicators**	**Challenge**	**Solution**
1.Data security policies from	1.1 Management planning	1.1.1 Hospital policies	Continuous updates and adherence to policies in real-time applications	Implement a flexible management plan with regular updates and agile methods

Level 1 indicators	Level 2 indicators	Level 3 indicators	Challenge	Solution
management department	1.2 Organizational structure	1.2.1 Leadership responsibility	Responsiveness of leadership and organizational systems in real-time scenarios	Establish a real-time incident response team with clear leadership and communication protocols
2.Trauma care data management from clinical department	2.1 Data capture	2.1.1 Operational safety of medical personnel	Secure data capture without disrupting workflow	Develop user-friendly security protocols and provide comprehensive training
		2.1.2 Dedicated responsibility system	Clear responsibilities in a fast-paced environment	Create detailed role-based access controls and automate responsibility assignments

Level 1 indicators	Level 2 indicators	Level 3 indicators	Challenge	Solution
		2.1.3 Data capture environment security	Securing physical and digital data capture environments	Utilize secure network configurations, regular audits, and environmental controls
		2.1.4 Data capture security training	Keeping training relevant and up-to-date	Offer continuous education and regular updates on security practices
		2.1.5 Data capture security knowledge	Ensuring staff have necessary security knowledge	Implement ongoing education programs and integrate security knowledge assessments
	2.2 Data storage	2.21 Data storage mode<br>2.22 Data storage sites	Securing various storage modes and locations	Employ end-to-end encryption and regular audits

Level 1 indicators	Level 2 indicators	Level 3 indicators	Challenge	Solution
		2.2.3 Mobile media identification	Managing security of data on mobile devices	Utilize mobile device management solutions
		2.2.4 Data storage security training	Comprehensive training and strict access controls	Regularly update training programs and use multifactor authentication
	2.3 Data transmission	2.3.1 Data transmission system<br>2.3.2 Dedicated responsibility system<br>2.3.3 Data transmission security training<br>2.3.4 Data transmission security operations	Protecting data during transmission and ensuring staff are trained	Implement secure communication protocols and conduct regular training

Level 1 indicators	Level 2 indicators	Level 3 indicators	Challenge	Solution
		2.3.5 Encrypted transmission of business systems<br>2.3.6 Remote O&M channel security	Ensuring encryption and secure remote operations	Use robust encryption standards and secure virtual private networks
	2.4 Data application	2.4.1 Data access hierarchy<br>2.4.2 Data application security training	Maintaining a secure and efficient access hierarchy	Implement fine-grained access controls and conduct regular training
		2.4.3 Approval of data usage results<br>2.4.4 Availability/accessibility	Ensuring data usage approval and accessibility	Establish strict approval workflows and use high availability systems
	2.5 Data destruction	2.5.1 Operational safety of medical personnel	Safely destroying data while maintaining compliance	Use secure deletion methods and provide comprehensive training

Level 1 indicators	Level 2 indicators	Level 3 indicators	Challenge	Solution
		2.5.2 Data destruction security training		
3. Information security technologies from the IT department	3.1 Information system partnership management	3.1.1 Vendor qualifications<br>3.1.2 Vendor confidentiality agreement	Ensuring vendors meet security requirements	Conduct thorough vetting processes and enforce stringent confidentiality agreements
	3.2 Information system monitoring and auditing	3.2.1 Monitoring and warning mechanism	Continuous monitoring in real-time applications	Implement automated monitoring tools and real-time alert systems
		3.2.2 System security audits	Performing regular, comprehensive security audits	Schedule periodic audits and use automated tools
	3.3 Information system environment security	3.3.1 Monitoring facilities<br>3.3.2 Safety protection facilities	Securing physical and digital environments	Utilize advanced surveillance systems and physical security measures

Level 1 indicators	Level 2 indicators	Level 3 indicators	Challenge	Solution
	3.4 Information system technology security	3.4.1 Automatic identification and processing of sensitive data	Identifying and securing sensitive data automatically	Implement machine learning algorithms and data loss prevention tools
		3.4.2 Data security protection technologies	Protecting data and ensuring reliable backups	Use state-of-the-art encryption, regular backup schedules, and redundancy
		3.4.3 System backup and restoration		
		3.4.4 System operations log	Maintaining comprehensive logs for security monitoring	Implement centralized logging systems
